# Circuit training on oxidative stress and arterial health: a health promotion perspective for obese adult men

**DOI:** 10.3389/fpubh.2025.1562193

**Published:** 2025-06-12

**Authors:** Woo-Hyeon Son, Yi-Sub Kwak, Min-Seong Ha

**Affiliations:** ^1^Design Institute, Inje University, Gimhae, Republic of Korea; ^2^Division of Navigation Convergence Studies, Korea Maritime & Ocean University, Busan, Republic of Korea; ^3^Department of Physical Education, Dong-Eui University, Busan, Republic of Korea; ^4^Laboratory of Sports Conditioning, Nutrition Biochemistry and Neuroscience, Department of Sport Science, College of Arts and Sports, University of Seoul, Seoul, Republic of Korea

**Keywords:** antioxidant, cardiovascular disease, circuit training, obesity, oxidative stress

## Abstract

**Background:**

Obesity leads to increased oxidative stress, disruption of the antioxidant system, and decreased bioavailability of nitric oxide (NO). This, in turn, contributes to impaired endothelial function. The resulting increase in arterial stiffness (AS) has been associated with an increased risk of cardiovascular disease (CVD). Regular physical activity improves the antioxidant system and vascular function. Circuit training combines aerobic exercise and resistance training, encapsulating the benefits of both types of exercise, and helps improve vascular function. We aimed to investigate the effects of circuit training on total oxidant status (TOS), total antioxidant status (TAS), NO, and atherosclerosis in adult men with obesity.

**Methods:**

A total of 25 obese men were randomly assigned to control (*n* = 12) or exercise groups (*n* = 13). The exercise group participated in circuit training three times per week for 12 weeks at an intensity corresponding to 60–80% of heart rate reserve (HRR). Anthropometrics, TOS, TAS, oxidative stress index (OSI), NO, and brachial-ankle pulse wave velocity (baPWV) were measured before and after the 12-week intervention.

**Results:**

Body mass index (BMI) (*p* < 0.001), TAS (*p* < 0.001), OSI (*p* < 0.05), NO (*p* < 0.05), and baPWV (L, R) (*p* < 0.05) values improved significantly in the exercise group following the 12-week intervention, while TOS values did not demonstrate a significant change. Furthermore, no change was observed in the control group.

**Conclusion:**

Our findings reveal that circuit training leads to improvements in BMI, TAS, OSI, NO, and baPWV in men with obesity, suggesting that it may contribute to an improvement in the antioxidant system and the prevention of CVD in obese men.

## Introduction

Obesity is defined as the accumulation of excessive fat due to physical inactivity, unhealthy eating patterns, and other factors, posing a serious public health problem worldwide (1, 2).

The increasing global prevalence of obesity among young adults is of particular concern (3), with rates among adults aged 20–39 years reported at 35.5% in the United States (4), 33.0% for men and 22.3% for women in Japan (5), and, as of 2021, 49.2% for men and 27.8% for women aged 20 years and older in South Korea, where the highest prevalence was observed to be 55.4% among men in their 30s (6).

Obesity is recognized as an independent risk factor for increased oxidative stress, which is defined as an imbalance between oxidants and antioxidants (7, 8), as excessive fat accumulation induces a chronic inflammatory state through elevated secretion of pro-inflammatory cytokines such as TNF-*α*, IL-6, and MCP-1, along with enhanced activation of nicotinamide adenine dinucleotide phosphate (NADPH) oxidase, all of which contribute to the increased production of reactive oxygen species (ROS) (9, 10). Catoi et al. reported that obese individuals exhibited elevated levels of pro-oxidants (total oxidant status, TOS) compared with individuals with normal weight, noting that this increase in oxidative stress contributes to the development of various diseases, including diabetes, endothelial dysfunction, and cardiovascular disease (CVD) (11, 12).

Although the human body possesses an antioxidant system comprising both enzymatic and non-enzymatic antioxidants that counteract oxidative stress and maintain homeostasis (13), obesity has been shown to reduce antioxidant capacity, thereby increasing oxidative stress (14, 15), as evidenced by a lower antioxidants (total antioxidant status, TAS) in individuals with obesity compared with those with normal weight (8), as well as an inverse correlation between body fat percentage and antioxidant capacity (16).

In pathological conditions characterized by increased oxidative stress, dysfunction of endothelial nitric oxide synthase (eNOS), which is responsible for nitric oxide (NO) production (17) along with reduced NO bioavailability, results in endothelial dysfunction (18). This, in turn, leads to reduced arterial elasticity and increased arterial stiffness (AS), a primary contributor to elevated systolic blood pressure and pulse pressure (19, 20), thereby increasing the risk of cardiovascular disease (CVD) and mortality (21).

It has been confirmed that regular physical activity offers many benefits, including improvements in the antioxidant system (22) and vascular health (23). It has been proposed as a non-pharmacological treatment option. Multiple studies have previously reported an alleviation of oxidative stress (24), reflected in changes in TAS (25), NO (26), and AS (27) levels subsequent to participation in regular exercise training.

Among the various forms of exercise training, circuit training combines the benefits of both aerobic and resistance training (28). It has been proposed as a time-efficient method to improve body composition and strength, obviating the need for specialized equipment or facilities (29). Further, previous studies have demonstrated improvements in body composition and cardiovascular risk factors with circuit training (30, 31).

Although circuit training has been well-documented for its benefits in improving body composition and reducing cardiovascular risk factors, there is a distinct lack of research exploring its direct impact on key vascular health markers, specifically oxidative stress, NO production, and AS in obese men. This study was designed to rigorously assess the effects of a structured circuit training regimen on TOS, TAS, NO, and AS levels in a high-risk patient cohort comprising obese men in Korea. By focusing on these critical biomarkers, the present study sought to elucidate the physiological pathways through which circuit training may mitigate CVD risk. Anchored in a health intervention framework, this study aimed to generate actionable evidence that can inform both clinical practice and public health policies geared toward the prevention and management of obesity-related vascular complications. We hypothesized that circuit training would significantly enhance antioxidant defense systems and vascular function, thereby offering a potent, scalable exercise intervention for obese men.

## Methods

### Participants

**Figure 1:**
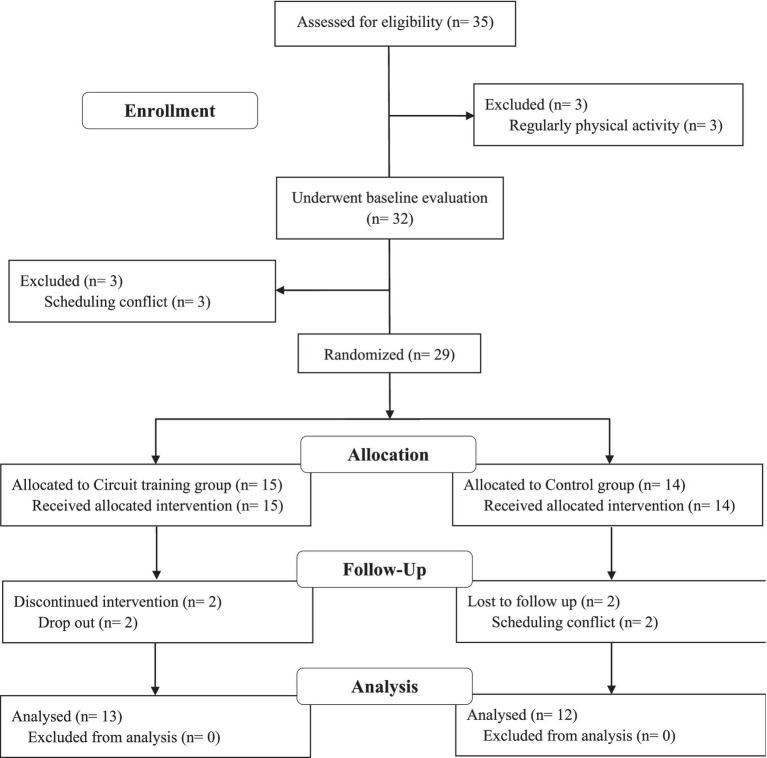
Diagram for the experimental study.

This study included 25 obese men (Body Mass Index [BMI] ≥ 25 kg/m^2^) (6) aged between 30 and 35 years, who had not engaged in regular physical activity in the past 6 months and had no history of diabetes, dyslipidemia, or CVD. All participants were randomly assigned to the control (CON, *n* = 12) or circuit training groups (EX, *n* = 13) (Figure 1). All study protocols were reviewed and approved by the Research Ethics Committee of Korea Maritime and Ocean University (Institutional Review Board Approval Number: KMOU IRB 2024–05) and conducted in accordance with the Declaration of Helsinki. Written informed consent was obtained from all participants prior to the study.

### Study design

After baseline measurements, participants were randomly assigned, using a parallel design, to either a non-exercise control group (CON, *n* = 12) or a circuit training group (EX, *n* = 13). Before and after the 12-week intervention period, all participants underwent blood sampling, AS assessment, and anthropometric evaluations. The EX group participated in a supervised circuit training program for 12 weeks, which included warm-up, main exercise (push-ups, squats, lunges, kickbacks, shoulder presses, pull-ups, hip-bridges, leg-raises, and crunches), and cool-down components. In contrast, the CON group did not engage in any structured physical activity and maintained their usual lifestyle.

### Anthropometrics

Height was measured without shoes using a portable stadiometer (InLabS50, InBody, Korea), and weight was measured in light clothing using an electronic scale (DB-1, CAS, South Korea). Body mass index (BMI) was computed as body weight in kilograms divided by height in meters squared (kg/m^2^).

### Blood sampling and analysis

Blood samples were collected from the antecubital vein using ethylenediaminetetraacetic acid tubes both before and after the 12-week circuit training program. Samples were centrifuged at 3,500 rpm for 10 min at 4°C, and the resulting plasma was stored at −70°C for subsequent analysis of TOS, TAS and NO levels. Serum TOS levels were determined using a commercial assay kit (Total Oxidant Status, Rel Assay Diagnostics, Gaziantep, Turkey) and measured spectrophotometrically using an automated analyzer (Beckman Coulter AU680, Tokyo, Japan) (32). Serum TAS levels were assessed using a Total Antioxidant Status kit (Rel Assay Diagnostics) with the same analyzer (33). NO levels were analyzed using a Total NO/Nitrite/Nitrate Assay kit (R&D Systems, Minneapolis, MN, United States), and absorbance was measured spectrophotometrically at 540 nm using a VERSAmax microplate reader (Molecular Devices, Sunnyvale, CA, USA) (34). The oxidative stress index (OSI) was calculated as TOS divided by TAS multiplied by 100 (OSI = TOS/TAS × 100) (35).

### Arterial stiffness

Participants arrived at the laboratory 30 min prior to the measurement. After lying in a supine position and resting for approximately 10 min to achieve maximal relaxation, AS was assessed using an automated vascular screening device (VP-1000 Plus, Omron Healthcare Co., Ltd., Kyoto, Japan) to determine baPWV (36). The measurement was performed by wrapping cuffs around both arms and ankles and attaching electrocardiogram electrodes to both wrists.

### Circuit training program

The design of the circuit training program was based on the exercise regimen proposed by Bocalini et al. (37) that has been modified for the present study. It consisted of 9 exercises: push-ups, squats, lunges, kickbacks, shoulder presses, pull-ups, hip-bridges, leg-raises, and crunches that were performed three times per week for 12 weeks. All participants were encouraged to complete each exercise within 60 s and to rest for 50 s between sets, performing three sets of each exercise during weeks 1–6 and increasing to four sets during weeks 7–12. Exercise intensity was monitored using a wrist-worn heart rate monitor (Polar RS400sd, APAC, USA) to ensure that heart rate reached 60–80% of heart rate reserve (Table 1).

**Table 1:** Circuit training program.

Order	Exercise	Duration, min	Week	Intensity	Sets	Frequency
Warm-up	Static stretching	10				3 times/wk
Main exercise	Push-ups squats, lunges, kick backs	35	1–6	60-70%HRR	3 sets	
	shoulder presses pull-ups hip-bridges leg-raises crunches	45	7–12	70-80%HRR	4 sets	
Cool-down	Static stretching	10				

### Data analysis

SPSS Statistics software version 25.0 (IBM Corp, Armonk, New York, USA) was used to conduct all statistical analyses. The Shapiro–Wilk test was used to assess data normality, and the effects of the circuit training program on BMI, TOS, TAS, NO, and baPWV were identified using a two-way repeated measures analysis of variance with group (EX and CON) and time (pre- and post-12 week) as independent variables. When significant interactions were noted, paired t-tests were used for *post hoc* comparisons. Data are presented as mean ± standard deviation. Statistical significance was set as *p* < 0.05.

## Results

No participants reported any adverse events or unfavorable symptom effects resulting from a circuit training program. BMI, TOS, TAC, NO and cfPWV pre and post 12 weeks for CON and EX groups are presented (Tables 2–4). Participants in the EX group completed 95% of the circuit training program under the supervision of a professional. No statistically significant differences were observed between the groups in the pre-test measurements. We observed significant group versus time interactions for BMI (*F* = 48.714, *p* < 0.001), TAS (*F* = 23.000, *p* < 0.001), OSI (*F* = 5.133, *p* < 0.05,), NO (*F* = 6.723, *p* < 0.05), baPWV (L) (*F* = 6.000, *p* < 0.05), and baPWV (R) (*F* = 4.715, *p* < 0.05). BMI (from 26.84 ± 1.57 to 25.77 ± 1.64 kg/m^2^, *t* = 9.298, *p* < 0.001) was significantly reduced in the EX group (Table 2). OSI (from 0.23 ± 0.04 to 0.20 ± 0.04, *t* = 3.049, *p* < 0.05), baPWV(L) (from 1263.38 ± 119.25 to 1222.46 ± 99.47 m/s, *t* = 2.210, *p* < 0.05), and baPWV(R) (from 1301.15 ± 99.47 to 1254.38 ± 87.71 m/s, *t* = 3.034, *p* < 0.05) were significantly reduced in the EX group (Tables 3, 4). TAS (from 1707.69 ± 122.69 to 1850.77 ± 130.03 μmol/L, *t* = −4.873, *p* < 0.001) was significantly elevated in the EX group (Table 3). NO (from 40.95 ± 7.05 to 44.71 ± 6.16 μmol/L, *t* = −2.702, *p* < 0.05) was significantly elevated in the EX group (Table 3).

**Table 2:** Characteristics of participants.

	CON (*n* = 12)			EX (*n* = 13)		
	Pre	Post	**Δ**	Pre	Post	**Δ**
Age, y	32.58 ± 1.73	**-**	**-**	32.15 ± 1.21	**-**	**-**
Height, cm	176.50 ± 3.55	**-**	**-**	177.23 ± 6.29	**-**	**-**
Weight, kg	83.97 ± 6.82	83.97 ± 7.72	0.0 ± 0.90	84.45 ± 8.45	81.06 ± 8.28	−3.39 ± 0.17
BMI, kg/m^2^	26.94 ± 1.90	26.94 ± 2.15	0.0 ± 0.52	26.84 ± 1.57	25.77 ± 1.64^***^	−1.07 ± 0.07^###^

Values are presented as mean (M) ± standard deviation (SD). BMI: body mass index; CON: control group; EX: exercise group. ^***^p < 0.001 different than Pre. ^###^ *p* < 0.001 different than CON.

**Table 3:** Change in TOS, TAS, OSI and NO pre and post in CON and EX group.

	CON (*n* = 12)			EX (*n* = 13)		
	Pre	Post	**Δ**	Pre	Post	**Δ**
TOS, μmol/L	3.90 ± 0.40	3.94 ± 0.53	0.40 ± 0.13	3.85 ± 0.54	3.59 ± 0.57	−0.26 ±0.03
TAS, μmol/L	1717.50 ± 152.62	1707.50 ± 143.37	10.00 ± 9.25	1707.69 ± 122.69	1850.77 ± 130.03^***^	143.08 ± 7.34^###^
OSI, arbitrary unit	0.23 ± 0.03	0.23 ± 0.04	0.00 ± 0.01	0.23 ± 0.04	0.20 ± 0.04^*^	−0.03 ± 0.00^#^
NO, μmol/L	42.70 ± 8.61	41.22 ± 7.51	−1.48 ± 1.10	40.95 ± 7.05	44.71 ± 6.16^*^	3.76 ± 0.89^#^

Values are presented as mean (M) ± standard deviation (SD). CON: control group; EX: exercise group; TOS: Total Oxidant Status; TAS; Total Antioxidant Status; NO: nitric oxide. ^*^p < 0.05, ***p < 0.001 different than Pre. ^#^*p* < 0.05, ###*p* < 0.001 different than CON.

**Table 4:** Change in baPWV pre and post in CON and EX group.

	CON (*n* = 12)			EX (*n* = 13)		
	Pre	Post	**Δ**	Pre	Post	**Δ**
baPWV (L), m/s	1263.42 ± 124.91	1295.00 ± 129.44	31.58 ± 4.53	1263.38 ± 119.25	1222.46 ± 99.47^*^	−40.92 ± 19.78^#^
baPWV(R), m/s	1295.25 ± 90.16	1320.17 ± 85.03	24.92 ± 5.13	1301.15 ± 99.47	1254.38 ± 87.71^*^	−46.77 ± 11.76^#^

Values are presented as mean (M) ± standard deviation (SD). CON: control group; EX: exercise group; baPWV: Brachial-Ankle Pulse Wave Velocity. *p < 0.05 different than Pre. ^#^*p* < 0.05 different than CON.

## Discussion

This study was conducted to verify the hypothesis that circuit training would have a positive effect on post-exercise TOS, TAS, NO, and AS levels in obese men. Several notable findings were observed in this study. First, there was an increase in TAS and NO levels. Second, significant reductions were observed in OSI and baPWV values. These findings suggest that circuit training is a viable intervention for enhancing vascular function, as evidenced by improvements in TAS, OSI, NO, and AS in obese men.

### TOS

Obesity has been associated with increased oxidative stress (38), primarily due to obesity-induced mitochondrial dysfunction, which promotes the overproduction of reactive oxygen species (ROS), ultimately resulting in elevated oxidative stress (39).

Furukawa et al. reported that fat accumulation increases oxidative stress (9), and Cagnacci et al. (40) reported a positive correlation between abdominal obesity and oxidative stress. Increased oxidative stress has been demonstrated to contribute to various pathological events, including insulin resistance, diabetes, and cardiovascular complications (41, 42). It is recognized as a promoter of the development of endothelial dysfunction and CVDs (43). A reduction in oxidative stress has been identified as a means to enhance vascular function (44). Physical activity or exercise has been proposed as a potential intervention strategy to improve vascular health due to its ability to reduce oxidative stress (45). This was demonstrated in a study by Roh et al. (46) which reported that aerobic exercise effectively reduces oxidative stress in obese adults.

Rosety-Rodriguez et al. (25) reported a reduction in oxidative damage after 12 weeks of circuit training in adults with Down syndrome, attributing this outcome to an increase in antioxidant enzymes such as superoxide dismutase and catalase, as well as an improvement in redox balance, which led to a reduction in oxidative stress (45). However, Deminice et al. (47) reported no change in oxidative stress after an acute session of circuit training in healthy young men. These results were attributed to factors such as duration of exercise, exercise mode, and the exercise protocol (48).

Our findings revealed that oxidative stress levels did not demonstrate a statistically significant difference between the EX and CON groups; however, a statistical trend toward slightly lowered oxidative stress was observed in the EX group, suggesting that regular participation in circuit training may contribute to a statistically significant reduction in oxidative stress. However, the impact of exercise on oxidative stress remains controversial (49, 50), necessitating further research that encompasses varying parameters such as exercise intensity and duration.

### TAS

Obese individuals have higher levels of oxidative stress compared with those having a normal body weight (51), and this increase in oxidative stress disrupts the homeostasis of the antioxidant system (52). It has been established that an increase in body weight is associated with a reduction in the antioxidant capacity of plasma (53). Chrysohoou et al. (7) reported that markers of antioxidant defense exhibited an inverse correlation with body fat percentage and abdominal obesity. Furthermore, Catoi et al. (11) revealed that serum levels of TAS, a marker of overall antioxidant status encompassing both enzymatic and non-enzymatic antioxidants, were diminished in obese individuals compared with normal-weight individuals.

Willcox et al. (54) reported that lower levels of antioxidants were associated with increased oxidative stress, which contributed to lipid peroxidation and tissue damage, thereby adversely affecting vascular function and structure (55, 56), while elevated antioxidant levels helped prevent the accumulation of ROS and reduced the risk of CVD (57).

Regular physical activity has been reported to stimulate antioxidant enzyme activity (58). Attarzadeh Hosseini et al. (59) further demonstrated that both high-intensity interval training and moderate-intensity continuous exercise significantly enhanced total antioxidant capacity in overweight and obese women. Zhang et al. (60) reported an increase in antioxidant enzyme levels after 8 weeks of circuit training in high-school wrestlers. It has been suggested that oxidative stress is mitigated by a reduction in body fat mass and the activation of erythroid-related nuclear factor 2 and antioxidant-responsive elements (61).

Our current findings revealed a significant TAS reduction in the exercise group, corroborating previous studies. Additionally, the OSI value, which provides information about the interactions between the TOS and TAS ratio (62), displayed a significant reduction in the exercise group, suggesting that antioxidant activity predominates over oxidative processes (35).

Our data suggest that a reduction in BMI through circuit training may help improve antioxidant capacity and redox equilibrium in individuals with obesity.

### Nitric oxide

Increased oxidative stress has been demonstrated to increase endothelium-derived contractile factors and impair the activation of eNOS, which reduces NO production, a key regulator of endothelial function that involves the prevention of platelet aggregation and adhesion and vasorelaxation (63, 64). An animal study by DeMarco et al. reported that increased oxidative stress decreased NO bioavailability in mice that had gained weight from a Western diet (65). This decline in the production and bioavailability of NO has been linked to various health complications, including atherosclerosis, hypertension, and endothelial dysfunction (66, 67).

Conversely, regular physical activity has been suggested to increase the bioavailability of NO (68), as demonstrated by elevated NO levels following consistent exercise in our previous study (69), by Ghadery et al. (70) who reported an upregulation of eNOS after 6 weeks of high-intensity interval training in obesity-induced rats, and by Guzel et al. (71) who observed a significant increase in NO after a single bout of high-intensity circuit training in sedentary men, where this effect was speculated to result from the rise in shear force during physical exertion that activates the Akt/eNOS signaling pathway, thereby enhancing NO production (72).

Our present data showed a significant increase in NO in the exercise group, indicating that circuit training may contribute to the reduction of BMI and the increase of antioxidant capacity, thereby improving NO levels and endothelial dysfunction.

### Arterial stiffness

Obesity-induced oxidative stress reduces the activation of eNOS, leading to decreased NO bioavailability, endothelial dysfunction, and vascular hypertrophy, which ultimately contribute to increased AS (73, 74), resulting in structural vascular changes, including alterations in elasticity, capacitance (75), and resistance that are associated with a higher incidence and mortality of CVD (76).

Safar et al. reported that the risk of AS increases in individuals with obesity, regardless of ethnicity, age, or blood pressure (77), and a cohort study by Ohkuma et al. (78) demonstrated that each 1 m/s increase in baPWV is associated with an approximately 12% higher risk of CVD. However, a reduction in BMI through increased and regular physical activity has been shown to improve vascular function (79). A study by Vlachopoulos et al. (80) reported that a 1 m/s reduction in PWV was associated with an approximately 7% reduction in CVD risk. Previous studies have reported a decrease in baPWV following twice-weekly circuit training in older adult women (81) and following 12 weeks of circuit training in postmenopausal women with hypertension (82). This indicates that the rise in NO, a vasodilator, resulting from exercise leads to a reduction in AS (83, 84).

Our present findings revealed a substantial reduction in AS in the exercise group, which was consistent with previous reports. Our data suggest that a reduction in BMI achieved through circuit training may lead to an increase in total antioxidant capacity and a subsequent rise in NO levels, which together may improve AS and help prevent CVD.

However, this study has several methodological limitations. First, dietary habits, particularly sodium intake, were not controlled, which may be associated with AS (85). Second, the daily routines of the study participants, such as physical activity and smoking, were not monitored, which may have influenced the potential effectiveness of the circuit training. Third, since the participants were obese men in their 30s, the findings may not be generalizable to broader populations, including women and individuals of different age groups. Fourth, genetic and epigenetic factors, which can affect individual responses to circuit training in relation to oxidative stress and arterial health, were not considered. Fifth, psychosocial factors such as motivation and mental well-being, which could influence adherence to circuit training programs, were also not assessed. Finally, the sample size was limited to 25 participants. Further studies with larger sample sizes that incorporate dietary, lifestyle, genetic, and psychosocial factors are warranted to substantiate and expand upon the findings of this study.

## Conclusion

This study investigated the effects of circuit training on oxidative stress, NO levels, and AS in obese men. Our results showed significant increases in TAS and NO levels, alongside improvements in OSI and baPWV, indicating that circuit training is an effective strategy for enhancing vascular health and reducing CVD risk in this population. These findings contribute to the existing literature by providing new evidence on the benefits of circuit training, with relevance for both developed and developing countries where obesity remains a growing health concern. The study underscores the importance of incorporating structured exercise programs into public health initiatives and clinical practice to manage oxidative stress and AS. We recommend further research with diverse populations and varied training protocols to confirm and extend these findings and encourage policymakers to adopt circuit training as part of more comprehensive obesity and cardiovascular health strategies.

## Data Availability

The original contributions presented in the study are included in the article/supplementary material, further inquiries can be directed to the corresponding author.

## References

[1] Martínez-Martínez E Cachofeiro V. Oxidative stress in obesity. Antioxidants. (2022) 11:–639. doi: 10.3390/antiox11040639 PMC9032430 35453323

[2] Tiwari A Balasundaram P Public health considerations regarding obesity. Treasure Island, FL: StatPearls Publishing. (2021). 34283488

[3] Katsoulis M Lai AG Diaz-Ordaz K Gomes M Pasea L Banerjee A. Identifying adults at high-risk for change in weight and BMI in England: a longitudinal, large-scale, population-based cohort study using electronic health records. Lancet Diabetes Endocrinol. (2021) 9:681–94. doi: 10.1016/S2213-8587(21)00207-2, PMID: 34481555 PMC8440227

[4] CDC. Obesity and severe obesity prevalence in adults: United States, august 2021–august 2023 Center for Disease Control and Prevention. Atlanta, GA: U.S. (2024).

[5] Iwase Y Hosokawa R. Associations between health interest scale dimensions and obesity risk: a cross-sectional study among Japanese employees. JMA J. (2025) 8:453–64. doi: 10.31662/jmaj.2024-0388, PMID: 40416019 PMC12095852

[6] Jeong SM Jung JH Yang YS Kim W Cho IY Lee YB. 2023 obesity fact sheet: prevalence of obesity and abdominal obesity in adults, adolescents, and children in Korea from 2012 to 2021. J Obes Metab Syndr. (2024) 33:27–35. doi: 10.7570/jomes24012 38531533 PMC11000515

[7] Chrysohoou C Panagiotakos DB Pitsavos C Skoumas I Papademetriou L Economou M. The implication of obesity on total antioxidant capacity in apparently healthy men and women: the ATTICA study. Nutr Metab Cardiovasc Dis. (2007) 17:590–7. doi: 10.1016/j.numecd.2006.05.007, PMID: 16901682

[8] Chen S Sun L Gao H Ren L Liu N Song G. Visfatin and oxidative stress influence endothelial progenitor cells in obese populations. Endocr Res. (2015) 40:83–7. doi: 10.3109/07435800.2014.952016, PMID: 25207957

[9] Furukawa S Fujita T Shimabukuro M Iwaki M Yamada Y Nakajima Y. Increased oxidative stress in obesity and its impact on metabolic syndrome. J Clin Invest. (2004) 114:1752–61. doi: 10.1172/JCI21625, PMID: 15599400 PMC535065

[10] den L Omer M Goodspeed L Wang S Wietecha T O’Brien K. Adipocyte-specific deficiency of NADPH oxidase 4 delays the onset of insulin resistance and attenuates adipose tissue inflammation in obesity. Arterioscler Thromb Vasc Biol. (2017) 37:466–75. doi: 10.1161/ATVBAHA.116.308749 28062496 PMC5323321

[11] Catoi AF Parvu A Galea RF Pop ID Muresan A Catoi C. Nitric oxide, oxidant status and antioxidant response in morbidly obese patients: the impact of 1-year surgical weight loss. Obes Surg. (2013) 23:1858–63. doi: 10.1007/s11695-013-0968-1, PMID: 23625335

[12] Niki E. Free radicals in the 1900's: from in vitro to in vivo. Free Radic Res. (2000) 33:693–704. doi: 10.1080/10715760000301221, PMID: 11237092

[13] Gonenc S Acikgoz O Semin I Ozgonul H. The effect of moderate swimming exercise on antioxidant enzymes and lipid peroxidation levels in children. Indian J Physiol Pharmacol. (2000) 44:340–4. PMID: 10941624

[14] Khutami C Sumiwi SA Khairul Ikram NK Muchtaridi M. The effects of antioxidants from natural products on obesity, dyslipidemia, diabetes and their molecular signaling mechanism. Int J Mol Sci. (2022) 23:2056. doi: 10.3390/ijms23042056, PMID: 35216172 PMC8875143

[15] Vona R Gambardella L Cittadini C Straface E Pietraforte D. Biomarkers of oxidative stress in metabolic syndrome and associated diseases. Oxidative Med Cell Longev. (2019) 2019:8267234. doi: 10.1155/2019/8267234, PMID: 31191805 PMC6525823

[16] Nono Nankam PA Nguelefack TB Goedecke JH Bluher M. Contribution of adipose tissue oxidative stress to obesity-associated diabetes risk and ethnic differences: focus on women of African ancestry. Antioxidants. (2021) 10:622. doi: 10.3390/antiox10040622 33921645 PMC8073769

[17] Li H Forstermann U. Uncoupling of endothelial NO synthase in atherosclerosis and vascular disease. Curr Opin Pharmacol. (2013) 13:161–7. doi: 10.1016/j.coph.2013.01.006, PMID: 23395155

[18] Forstermann U Xia N Li H. Roles of vascular oxidative stress and nitric oxide in the pathogenesis of atherosclerosis. Circ Res. (2017) 120:713–35. doi: 10.1161/CIRCRESAHA.116.309326, PMID: 28209797

[19] Mitchell GF Hwang SJ Vasan RS Larson MG Pencina MJ Hamburg NM. Arterial stiffness and cardiovascular events: the Framingham heart study. Circulation. (2010) 121:505–11. doi: 10.1161/CIRCULATIONAHA.109.886655, PMID: 20083680 PMC2836717

[20] Mozos I Malainer C Horbanczuk J Gug C Stoian D Luca CT. Inflammatory markers for arterial stiffness in cardiovascular diseases. Front Immunol. (2017) 8:1058. doi: 10.3389/fimmu.2017.01058, PMID: 28912780 PMC5583158

[21] Zhong Q Hu MJ Cui YJ Liang L Zhou MM Yang YW. Carotid-femoral pulse wave velocity in the prediction of cardiovascular events and mortality: an updated systematic review and Meta-analysis. Angiology. (2018) 69:617–29. doi: 10.1177/0003319717742544, PMID: 29172654

[22] Meng Q Su CH. The impact of physical exercise on oxidative and nitrosative stress: balancing the benefits and risks. Antioxidants. (2024) 13:573. doi: 10.3390/antiox13050573, PMID: 38790678 PMC11118032

[23] Hasegawa N Fujie S Kurihara T Homma T Sanada K Sato K. Effects of habitual aerobic exercise on the relationship between intramyocellular or extramyocellular lipid content and arterial stiffness. J Hum Hypertens. (2016) 30:606–12. doi: 10.1038/jhh.2016.28, PMID: 27169824

[24] Sampaio RC Moura NR Barros MP Hatanaka E Priviero FBM Moraes C. Twice-weekly exercise training reduces oxidative stress and proinflammatory cytokine levels in elder women. Motriz Rev Educ Fis. (2019) 25:e101990. doi: 10.1590/s1980-6574201900030001

[25] Rosety-Rodriguez M Bernardi M Elosegui S Rosety I Diaz AJ Rosety MA. A short-term resistance training circuit improved antioxidants in sedentary adults with down syndrome. Oxidative Med Cell Longev. (2021) 2021:8811153. doi: 10.1155/2021/8811153, PMID: 33532037 PMC7840230

[26] Arefirad T Seif E Sepidarkish M Mohammadian Khonsari N Mousavifar SA Yazdani S. Effect of exercise training on nitric oxide and nitrate/nitrite (NOx) production: a systematic review and meta-analysis. Front Physiol. (2022) 13:953912. doi: 10.3389/fphys.2022.953912, PMID: 36267589 PMC9576949

[27] Michalski AC Ferreira AS Midgley AW Costa VAB Fonseca GF da Silva NSL. Mixed circuit training acutely reduces arterial stiffness in patients with chronic stroke: a crossover randomized controlled trial. Eur J Appl Physiol. (2023) 123:121–34. doi: 10.1007/s00421-022-05061-8, PMID: 36205814

[28] Figueroa A Park SY Seo DY Sanchez-Gonzalez MA Baek YH. Combined resistance and endurance exercise training improves arterial stiffness, blood pressure, and muscle strength in postmenopausal women. Menopause. (2011) 18:980–4. doi: 10.1097/gme.0b013e3182135442, PMID: 21540753

[29] Marcos-Pardo PJ Orquin-Castrillon FJ Gea-Garcia GM Menayo-Antunez R Gonzalez-Galvez N Vale RGS. Effects of a moderate-to-high intensity resistance circuit training on fat mass, functional capacity, muscular strength, and quality of life in elderly: a randomized controlled trial. Sci Rep. (2019) 9:7830. doi: 10.1038/s41598-019-44329-6, PMID: 31127163 PMC6534570

[30] Jung WS Kim YY Park HY. Circuit training improvements in Korean women with sarcopenia. Percept Mot Skills. (2019) 126:828–42. doi: 10.1177/0031512519860637, PMID: 31284844

[31] Jung WS Kim YY Kim JW Park HY. Effects of circuit training program on cardiovascular risk factors, vascular inflammatory markers, and insulin-like growth Factor-1 in elderly obese women with sarcopenia. Rev Cardiovasc Med. (2022) 23:134. doi: 10.31083/j.rcm2304134, PMID: 39076242 PMC11273982

[32] Erel O. A new automated colorimetric method for measuring total oxidant status. Clin Biochem. (2005) 38:1103–11. doi: 10.1016/j.clinbiochem.2005.08.008, PMID: 16214125

[33] Erel O. A novel automated method to measure total antioxidant response against potent free radical reactions. Clin Biochem. (2004) 37:112–9. doi: 10.1016/j.clinbiochem.2003.10.014, PMID: 14725941

[34] Giovannoni G Land JM Keir G Thompson EJ Heales SJ. Adaptation of the nitrate reductase and Griess reaction methods for the measurement of serum nitrate plus nitrite levels. Ann Clin Biochem. (1997) 34:193–8. doi: 10.1177/000456329703400212 9133256

[35] Sanchez-Rodriguez MA Mendoza-Nunez VM. Oxidative stress indexes for diagnosis of health or disease in humans. Oxidative Med Cell Longev. (2019) 2019:4128152. doi: 10.1155/2019/4128152, PMID: 31885788 PMC6899293

[36] Meyer ML Tanaka H Palta P Patel MD Camplain R Couper D. Repeatability of central and peripheral pulse wave velocity measures: the atherosclerosis risk in communities (ARIC) study. Am J Hypertens. (2016) 29:470–5. doi: 10.1093/ajh/hpv127, PMID: 26232036 PMC4850900

[37] Bocalini DS Pontes F Luksevicius R Nolasco R Serra A Rodriguez D. Effects of circuit-based exercise programs on the body composition of elderly obese women. Clin Interv Aging. (2012) 7:551–6. doi: 10.2147/CIA.S33893 23271901 PMC3526879

[38] Charradi K Elkahoui S Limam F Aouani E. High-fat diet induced an oxidative stress in white adipose tissue and disturbed plasma transition metals in rat: prevention by grape seed and skin extract. J Physiol Sci. (2013) 63:445–55. doi: 10.1007/s12576-013-0283-6, PMID: 24158847 PMC11812467

[39] Lin Y Berg AH Iyengar P Lam TK Giacca A Combs TP. The hyperglycemia-induced inflammatory response in adipocytes: the role of reactive oxygen species. J Biol Chem. (2005) 280:4617–26. doi: 10.1074/jbc.M411863200, PMID: 15536073

[40] Cagnacci A Cannoletta M Palma F Bellafronte M Romani C Palmieri B. Relation between oxidative stress and climacteric symptoms in early postmenopausal women. Climacteric. (2015) 18:631–6. doi: 10.3109/13697137.2014.999659, PMID: 25536006

[41] Jung RT. Obesity as a disease. Br Med Bull. (1997) 53:307–21. doi: 10.1093/oxfordjournals.bmb.a011615, PMID: 9246838

[42] Pi-Sunyer FX. Health implications of obesity. Am J Clin Nutr. (1991) 53:1595S–603S. doi: 10.1093/ajcn/53.6.1595S, PMID: 2031492

[43] Sharebiani H Keramat S Chavoshan A Fazeli B Stanek A. The influence of antioxidants on oxidative stress-induced vascular aging in obesity. Antioxidants. (2023) 12:295. doi: 10.3390/antiox12061295, PMID: 37372025 PMC10295268

[44] Wang S Hu S Mao Y. The mechanisms of vascular aging. Aging Med. (2021) 4:153–8. doi: 10.1002/agm2.12151, PMID: 34250433 PMC8251869

[45] El Assar M Alvarez-Bustos A Sosa P Angulo J Rodriguez-Manas L. Effect of physical activity/exercise on oxidative stress and inflammation in muscle and vascular aging. Int J Mol Sci. (2022) 23:713. doi: 10.3390/ijms23158713, PMID: 35955849 PMC9369066

[46] Roh HT So WY. The effects of aerobic exercise training on oxidant-antioxidant balance, neurotrophic factor levels, and blood-brain barrier function in obese and non-obese men. J Sport Health Sci. (2017) 6:447–53. doi: 10.1016/j.jshs.2016.07.006, PMID: 30356625 PMC6189263

[47] Deminice R Sicchieri T Mialich MS Milani F Ovidio PP Jordao AA. Oxidative stress biomarker responses to an acute session of hypertrophy-resistance traditional interval training and circuit training. J Strength Cond Res. (2011) 25:798–804. doi: 10.1519/JSC.0b013e3181c7bac6, PMID: 20581699

[48] Goto C Higashi Y Kimura M Noma K Hara K Nakagawa K. Effect of different intensities of exercise on endothelium-dependent vasodilation in humans: role of endothelium-dependent nitric oxide and oxidative stress. Circulation. (2003) 108:530–5. doi: 10.1161/01.CIR.0000080893.55729.28, PMID: 12874192

[49] Kawamura T Muraoka I. Exercise-induced oxidative stress and the effects of antioxidant intake from a physiological viewpoint. Antioxidants. (2018) 7:119. doi: 10.3390/antiox7090119, PMID: 30189660 PMC6162669

[50] Lu Z Xu Y Song Y Biro I Gu Y. A mixed comparisons of different intensities and types of physical exercise in patients with diseases related to oxidative stress: a systematic review and network Meta-analysis. Front Physiol. (2021) 12:700055. doi: 10.3389/fphys.2021.700055, PMID: 34421637 PMC8375596

[51] Vincent HK Bourguignon C Vincent KR. Resistance training lowers exercise-induced oxidative stress and homocysteine levels in overweight and obese older adults. Obesity (Silver Spring). (2006) 14:1921–30. doi: 10.1038/oby.2006.224, PMID: 17135607

[52] Amirkhizi F Siassi F Djalali M Shahraki SH. Impaired enzymatic antioxidant defense in erythrocytes of women with general and abdominal obesity. Obes Res Clin Pract. (2014) 8:e26–34. doi: 10.1016/j.orcp.2012.07.004, PMID: 24548574

[53] Karaouzene N Merzouk H Aribi M Merzouk SA Berrouiguet AY Tessier C. Effects of the association of aging and obesity on lipids, lipoproteins and oxidative stress biomarkers: a comparison of older with young men. Nutr Metab Cardiovasc Dis. (2011) 21:792–9. doi: 10.1016/j.numecd.2010.02.007, PMID: 20554180

[54] Willcox BJ Curb JD Rodriguez BL. Antioxidants in cardiovascular health and disease: key lessons from epidemiologic studies. Am J Cardiol. (2008) 101:75D–86D. doi: 10.1016/j.amjcard.2008.02.012, PMID: 18474278

[55] Yesilbursa D Serdar Z Serdar A Sarac M Coskun S Jale C. Lipid peroxides in obese patients and effects of weight loss with orlistat on lipid peroxides levels. Int J Obes. (2005) 29:142–5. doi: 10.1038/sj.ijo.0802794, PMID: 15467775

[56] Leopold JA Loscalzo J. Oxidative risk for atherothrombotic cardiovascular disease. Free Radic Biol Med. (2009) 47:1673–706. doi: 10.1016/j.freeradbiomed.2009.09.009, PMID: 19751821 PMC2797369

[57] Pham-Huy LA He H Pham-Huy C. Free radicals, antioxidants in disease and health. Int J Biomed Sci. (2008) 4:89–96. doi: 10.59566/IJBS.2008.4089, PMID: 23675073 PMC3614697

[58] Radak Z Zhao Z Koltai E Ohno H Atalay M. Oxygen consumption and usage during physical exercise: the balance between oxidative stress and ROS-dependent adaptive signaling. Antioxid Redox Signal. (2013) 18:1208–46. doi: 10.1089/ars.2011.4498, PMID: 22978553 PMC3579386

[59] Attarzadeh Hosseini SR Moazzami M Farahati S Bahremand M Sadegh Eghbali F. Effects of high-intensity interval training versus moderate-intensity continuous training on the total antioxidant capacity, malondialdehyde, and superoxide dismutase in obese/overweight middle-aged women. Iran J Endocrinol Metab. (2020) 22:207–13.

[60] Vincent HK Taylor AG. Biomarkers and potential mechanisms of obesity-induced oxidant stress in humans. Int J Obes. (2006) 30:400–18. doi: 10.1038/sj.ijo.0803177, PMID: 16302012

[61] Souza J Silva RA Luz Scheffer D Penteado R Solano A Barros L. Physical-exercise-induced antioxidant effects on the brain and skeletal muscle. Antioxidants. (2022) 11:826. doi: 10.3390/antiox11050826, PMID: 35624690 PMC9138070

[62] Soylu Karapinar O Pinar N Ozcan O Ozgur T Dolapcioglu K. Protective effect of alpha-lipoic acid in methotrexate-induced ovarian oxidative injury and decreased ovarian reserve in rats. Gynecol Endocrinol. (2017) 33:653–9. doi: 10.1080/09513590.2017.1306847, PMID: 28361557

[63] Kim JA Jang HJ Martinez-Lemus LA Sowers JR. Activation of mTOR/p70S6 kinase by ANG II inhibits insulin-stimulated endothelial nitric oxide synthase and vasodilation. Am J Physiol Endocrinol Metab. (2012) 302:E201–8. doi: 10.1152/ajpendo.00497.2011 22028412 PMC3340897

[64] Korita I Bulo A Langlois M Blaton V. Inflammation markers in patients with cardiovascular disease and metabolic syndrome. J Med Biochem. (2013) 32:214. doi: 10.2478/jomb-2013-0016

[65] DeMarco VG Habibi J Jia G Aroor AR Ramirez-Perez FI Martinez-Lemus LA. Low-dose mineralocorticoid receptor blockade prevents Western diet-induced arterial stiffening in female mice. Hypertension. (2015) 66:99–107. doi: 10.1161/HYPERTENSIONAHA.115.05674, PMID: 26015449 PMC4465849

[66] Loscalzo J. Nitric oxide insufficiency, platelet activation, and arterial thrombosis. Circ Res. (2001) 88:756–62. doi: 10.1161/hh0801.089861, PMID: 11325866

[67] Stamler JS Loh E Roddy MA Currie KE Creager MA. Nitric oxide regulates basal systemic and pulmonary vascular resistance in healthy humans. Circulation. (1994) 89:2035–40. doi: 10.1161/01.cir.89.5.2035, PMID: 7514109

[68] Barton M. Cholesterol and atherosclerosis: modulation by oestrogen. Curr Opin Lipidol. (2013) 24:214–20. doi: 10.1097/MOL.0b013e3283613a94, PMID: 23594711

[69] Son WM Sung KD Cho JM Park SY. Combined exercise reduces arterial stiffness, blood pressure, and blood markers for cardiovascular risk in postmenopausal women with hypertension. Menopause. (2017) 24:262–8. doi: 10.1097/GME.0000000000000765, PMID: 27779565

[70] Ghadery B Ghazalian F Hosseini SA Natanzy HA Shamsoddini A. The effect of six weeks of high intensity interval training on eNOS and PGC-1α gene expression in the heart tissue of male obese rats. Jundishapur J Health Sci. (2020) 12:e100280.

[71] Guzel NA Hazar S Erbas D. Effects of different resistance exercise protocols on nitric oxide, lipid peroxidation and creatine kinase activity in sedentary males. J Sports Sci Med. (2007) 6:417–22. PMID: 24149472 PMC3794479

[72] Tidball JG Wehling-Henricks M. Nitric oxide synthase deficiency and the pathophysiology of muscular dystrophy. J Physiol. (2014) 592:4627–38. doi: 10.1113/jphysiol.2014.274878, PMID: 25194047 PMC4253467

[73] Aroor AR Jia G Sowers JR. Cellular mechanisms underlying obesity-induced arterial stiffness. Am J Physiol Regul Integr Comp Physiol. (2018) 314:R387–98. doi: 10.1152/ajpregu.00235.2016, PMID: 29167167 PMC5899249

[74] Jia G Aroor AR DeMarco VG Martinez-Lemus LA Meininger GA Sowers JR. Vascular stiffness in insulin resistance and obesity. Front Physiol. (2015) 6:231. doi: 10.3389/fphys.2015.00231 26321962 PMC4536384

[75] Liao J Farmer J. Arterial stiffness as a risk factor for coronary artery disease. Curr Atheroscler Rep. (2014) 16:387. doi: 10.1007/s11883-013-0387-8, PMID: 24402301

[76] Stanek A Grygiel-Gorniak B Brozyna-Tkaczyk K Myslinski W Cholewka A Zolghadri S. The influence of dietary interventions on arterial stiffness in overweight and obese subjects. Nutrients. (2023) 15:1440. doi: 10.3390/nu15061440 36986170 PMC10058695

[77] Safar ME Czernichow S Blacher J. Obesity, arterial stiffness, and cardiovascular risk. J Am Soc Nephrol. (2006) 17:S109. doi: 10.1681/ASN.2005121321, PMID: 16565231

[78] Ohkuma T Ninomiya T Tomiyama H Kario K Hoshide S Kita Y. Brachial-ankle pulse wave velocity and the risk prediction of cardiovascular disease: an individual participant data meta-analysis. Hypertension. (2017) 69:1045–52. doi: 10.1161/HYPERTENSIONAHA.117.09097, PMID: 28438905

[79] Hill H Elliot CA Lizamore CA Hamlin MJ. Physical activity has a stronger correlation with arterial stiffness than strength, balance, or BMI in an older population. Front Aging. (2023) 4:1279479. doi: 10.3389/fragi.2023.1279479, PMID: 38162458 PMC10755870

[80] Vlachopoulos C Aznaouridis K Stefanadis C. Aortic stiffness for cardiovascular risk prediction: just measure it, just do it! J Am Coll Cardiol. (2014) 63:647–9. doi: 10.1016/j.jacc.2013.10.040, PMID: 24239659

[81] Miura H Takahashi Y Maki Y Sugino M. Effects of exercise training on arterial stiffness in older hypertensive females. Eur J Appl Physiol. (2015) 115:1847–54. doi: 10.1007/s00421-015-3168-y, PMID: 25869875

[82] Jeon K Lee S Hwang MH. Effect of combined circuit exercise on arterial stiffness in hypertensive postmenopausal women: a local public health center-based pilot study. Menopause. (2018) 25:1442–7. doi: 10.1097/GME.0000000000001154, PMID: 29975283

[83] Hasegawa N Fujie S Horii N Miyamoto-Mikami E Tsuji K Uchida M. Effects of different exercise modes on arterial stiffness and nitric oxide synthesis. Med Sci Sports Exerc. (2018) 50:1177–85. doi: 10.1249/MSS.0000000000001567, PMID: 29381650

[84] Kim HK Hwang CL Yoo JK Hwang MH Handberg EM Petersen JW. All-extremity exercise training improves arterial stiffness in older adults. Med Sci Sports Exerc. (2017) 49:1404–11. doi: 10.1249/MSS.0000000000001229, PMID: 28166118 PMC5474160

[85] Safar ME Temmar M Kakou A Lacolley P Thornton SN. Sodium intake and vascular stiffness in hypertension. Hypertension. (2009) 54:203–9. doi: 10.1161/HYPERTENSIONAHA.109.129585, PMID: 19581511

